# Metals Induce Genotoxicity in Three Cardoon Cultivars: Relation to Metal Uptake and Distribution in Extra- and Intracellular Fractions

**DOI:** 10.3390/plants11040475

**Published:** 2022-02-09

**Authors:** Maria Cristina Sorrentino, Simonetta Giordano, Fiore Capozzi, Valeria Spagnuolo

**Affiliations:** Department of Biology, Campus Monte S. Angelo, University of Naples Federico II, Via Cinthia 4, 80126 Napoli, Italy; mariacristina.sorrentino@unina.it (M.C.S.); giordano@unina.it (S.G.); valeria.spagnuolo@unina.it (V.S.)

**Keywords:** *Cynara cardunculus*, EDTA washing, ISSR, Cadmium, Lead

## Abstract

Heavy metal-polluted soil represents an important stress condition for plants. Several studies demonstrated that growth inhibition under metal stress and metal-induced damages, including genotoxicity, is particularly pronounced at the early stages of seedling growth. Moreover, it is reported that heavy metals enter the cytoplasm to exert their detrimental effect, including DNA damage. In this work, we estimated (i) metal-induced genotoxicity by ISSR molecular markers and (ii) the distribution of the metal fractions between symplast and apoplast by EDTA washing, in three cultivars of *Cynara cardunculus* var. *altilis* (L.) DC (Sardo, Siciliano, and Spagnolo), grown in hydroponics for 15 days with Cd or Pb: In line with the literature, in all cultivars, the genotoxic damage induced by Pb was more severe compared to Cd. However, a cultivar-specific response was evidenced since Spagnolo showed, under metal stress, a significantly higher genome template stability compared to the other examined cultivars. The lower genotoxicity observed in Spagnolo could depend on the lower intracellular metal concentration measured in this cultivar by chemical analysis. Accordingly, light microscopy highlighted that Spagnolo developed smaller and more numerous epidermal cells under metal stress; these cells would provide a larger wall surface offering a wider metal sequestration compartment in the apoplast.

## 1. Introduction

Plants are sessile organisms; therefore, they cannot escape from environmental stresses, being constantly exposed to both abiotic and biotic agents, which can frequently induce oxidative and genotoxic damage. Abiotic stresses include unfavorable growth conditions, such as water, salt, and temperature stresses, and a multitude of xenobiotic substances spread in the environment, such as organic pollutants and heavy metals. All the d-block elements of the periodic table have been identified as heavy metals, based on their density higher than 5 g cm^−3^ [1]. Due to their specific properties, heavy metals are employed in a wide variety of industrial applications. Consequently, geogenic, agricultural, pharmaceutical, and domestic wastes, as well as atmospheric sources continuously release heavy metals to the environment. For example, the estimated annual Pb production is 15,000 million tons followed by Cd (22,000 tons) [2].

Plants require only 19 elements for their metabolic functions, including the macronutrients C, O, H, Mg, S, N, Ca, P, and K and micronutrients Cu, Zn, Mn, Fe, Mo, B, Ni, Co, Cl, and Br [1]. These elements allow for all physiological and biochemical processes in the plant cell, such as pigment biosynthesis, photosynthesis, nucleic acid, protein, and carbohydrate metabolism. Remarkably, while some heavy metals, such as Cu and Zn, are used as microelements, others, such as Cd, Cr, Pb, and Hg generate toxic effects for plants, e.g., leaf chlorosis and necrosis, low biomass production, photosynthesis decline, water stress, and altered nutrient assimilation [3,4].

Lead is considered as one of the most toxic heavy metals, diffuse in the environment on a global scale [5,6,7]. When exposed to this metal, even at low concentrations, e.g., [8], plants usually experience dangerous genotoxic effects, such as mitosis disturbance, micronucleus induction, and DNA damage [9,10], as well as cyto-physiological alterations affecting membrane permeability and enzymatic activities [7,11]. Among heavy metals, cadmium is well known for its severe genotoxic effect; specifically, in plants, Cd can induce chromosomal aberrations and micronucleus formation [12]. Cadmium can substitute for Zn in the zinc-finger motif of the transcription factors in some DNA-binding proteins [13]; it can also bind adenine and guanine molecules in the DNA strand altering the double helix structure [14].

Heavy metal-polluted soil represents one of the most important stress conditions for plants. However, the possibility to induce genotoxic responses in plants is closely dependent on the bioavailability of the toxicants, the form in which they come into contact with the root tissues, and the amount of toxicant absorbed and translocated to specific target tissues; under this aspect, the use of hydroponics allows us to estimate genotoxicity induced by known doses of metals, supplied in bioavailable form.

Among experimental approaches to detect genotoxicity, multi-locus molecular markers provide an easy and fast method, since they do not require sequence information. Particularly, Inter Simple Sequence Repeats (ISSR), used in the present work, are more reliable than Random Amplified Polymorphic DNA (RAPD) because they use longer primers and higher annealing temperatures compared to the latter [15]. Several studies have demonstrated that growth inhibition under heavy metal stress in plants is particularly pronounced during seed germination at the early stages of seedling growth, e.g., [1,16] since the competition between heavy metal ions and the essential nutrient cations for binding and absorption by the root surface seem more active at the early growth stages. This suggests that the genotoxic effect induced by heavy metals could be more pronounced at the early steps of plant development.

In previous works, we found that *Cynara cardunculus* cv “Spagnolo” could face Cd and Pb metal stress with multiple responses that overall safeguarded plant growth and cell structure and functions, compared to other two cultivars (“Sardo” and “Siciliano”). According to previous data, we hypothesized that *C. cardunculus* cv. Spagnolo could cope with the toxicity induced by metals more effectively than the other two cultivars. In the present work, to verify if *C. cardunculus* cv Spagnolo had specific ability to face metal-induced genotoxic damage, we estimated in plants of *C. cardunculus* cv. Sardo, Siciliano, and Spagnolo, grown in hydroponics with known concentrations of Cd or Pb: (i) genotoxicity by ISSR molecular markers; (ii) the distribution of the metal fractions between symplast and apoplast by EDTA washing.

## 2. Results

### 2.1. DNA Variability and Genotoxic Effect

The 20 primers used provided different levels of variance (Table S1). In particular, 10, MAN, and TE showed low variability, although not always homogeneous over all cultivars. For example, TE showed no variance in Spagnolo, where no different bands were produced (i.e., a, b, c, and d), but the same primer induced some changes in the banding profile in the other two cultivars. As for primers exhibiting high level of variance, in Sardo, the most variable banding pattern was provided by the primer w898, inducing 32 band variations with Cd treatment and 40 with Pb. Similarly, in Siciliano, primer w898 induced considerable variance of the banding profile with both metals (27 and 28, with Cd and Pb, respectively). As for the Spagnolo, the primers mostly contributing to the variance were 22, 23, and w843; the other primers produced a relatively low level of variance compared to the other cultivars, or even no variance (see banding variation with the primers 15, 18, 19, and 20), resulting in a higher GTS calculated for Spagnolo under metal stress.

Regarding the genotoxic effect induced by the single metal, Pb appeared more harmful than Cd, at least in Sardo and Siciliano, with 334 and 233 band variations, respectively, observed under Pb stress, vs. 281 and 221 band variations evidenced under Cd stress. In Spagnolo the two heavy metals showed a lower and comparable genotoxic effect, with 174 band changes in the presence of Cd and 173 in the presence of Pb.

**Figure 1:**
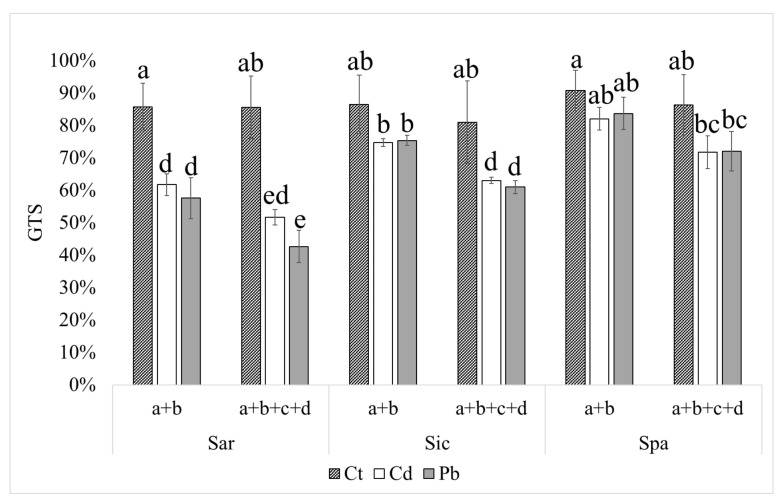
Average genome template stability (GTS, expressed as percentage) and standard deviation (*n* = 3) in control (Ct), cadmium treated (Cd), and lead treated (Pb) cardoon cultivars Sardo (Sar), Siciliano (Sic), and Spagnolo (Spa). Different letters indicate significant differences according to Tukey’s test, *p* < 0.05.

Considering the variation over all primers within each treatment (Figure 1), DNA variability was approximately 10–20% in all the three cultivars in control conditions (metal-untreated cultures); therefore, a genome stability of approximately 80% represented the threshold below which genotoxic damage could be assumed to occur. Both Cd and Pb induced a significant decrease in the genome template stability, especially evident in Sardo (with a decrease of approximately 40% in Pb-treated samples compared to control), and secondly in Siciliano cardoon, but to the limit of statistical significance in Spagnolo (Figure 1).

### 2.2. Chemical Analyses

Chemical analysis of the plant tissue (Table 1) highlighted that all the tested cultivars were able to accumulate Cd and Pb supplied in the culture medium, with significant higher concentrations in Sardo, mainly in roots. Metal concentration found in EDTA-washed leaves (representing the intra-cellular metal fraction) was lower in Spagnolo, in comparison with the other cultivars, although significantly lower only in the presence of Cd. For both metals, the concentrations measured in the roots (R) were significantly higher than those measured in the leaves (L); accordingly, translocation factors (TF = (L)/(R)) were lower than 1; nevertheless, for Cd the translocation factor was one order of magnitude higher than Pb. Moreover, TF values showed a similar trend in the three tested cultivars.

### 2.3. Leaf Epidermal Cell: Sizing and Counting

**Figure 2:**
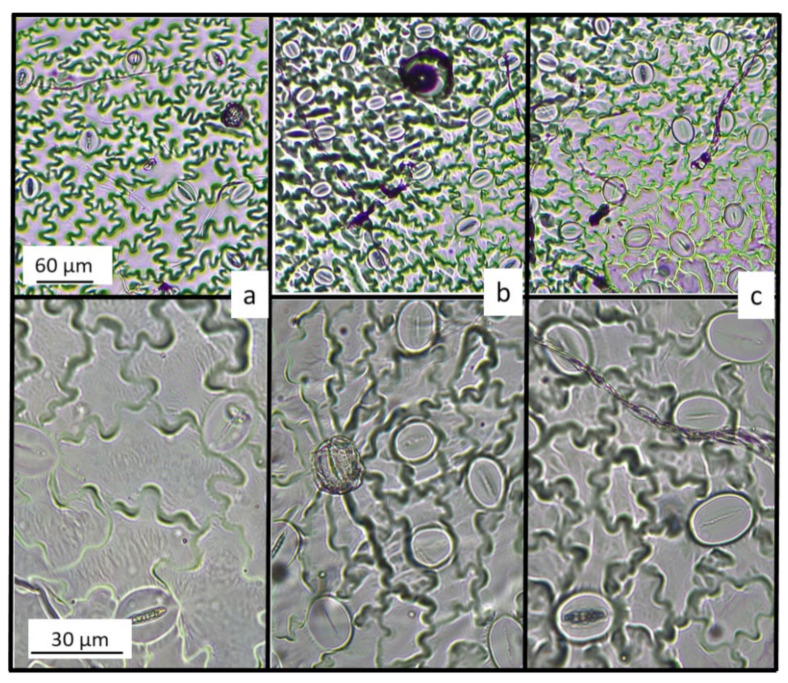
Leaf abaxial surface of C. cardunculus cv. Spagnolo; (**a**) control; (**b**) Cd-treated sample; (**c**) Pb-treated sample.

Epidermal cells had sinuous profiles in all cultivars and treatments; cells also had irregular shape; some were isodiametric, others elongated (for cv Spagnolo, see Figure 2). Specifically, elongated cells were frequently observed in all control leaves and in all treated leaves of Sardo and Siciliano; by contrast, isodiametric epidermal cells were frequently observed in Spagnolo under metal stress. The results of ImageJ analyses (Table 2) highlighted non-significant differences between Sardo and Siciliano in all treatments. In contrast, a significant decrease in the cell size was observed in Spagnolo under metal stress, where average cell area roughly halved compared to control (Table 2; Figure 2). As a consequence, the number of cells (Table 2 and Figure S1) significantly increased in the epidermal layer in the cultivar Spagnolo grown with Cd and Pb treatments.

## 3. Discussion

Among the different techniques used to study genotoxicity, ISSR molecular markers, here applied, represent a useful tool for quantitative comparisons in contrast to other approaches, such as comet assay and micronuclei observation, which give only qualitative data [17]. These techniques need to be repeated to provide reliable results, since they are grounded on the electrophoresis of a single nucleus or the observations of a few cells, respectively. Moreover, unlike the multilocus approach (i.e., ISSR), they only detect the genotoxic damage when DNA strand ruptures occur [18].

Differently from animal cells, plant cell environment is composed of two distinct compartments: the apoplast, including the extracellular spaces and the cell walls, and the symplast, comprising all protoplasts connected via plasmodesmata [19]. The literature contributions focused on metal toxicity in plant cells underline that heavy metals enter the intracellular environment and exert their detrimental effects, including genotoxic damage, e.g., [3,20,21,22]. Although in the last 20 years the interest of the scientific community about the genotoxic effects due to heavy metals in plants has increased, this is the first work exploring the possible relationship between the genotoxic damage induced by Cd and Pb and their distribution in the apoplast and symplast. Our results showed that both metals entered the cytoplasm of the three cultivars here examined, but Sardo showed an accumulation of Cd and Pb significantly higher than the other two cultivars, especially in the roots. In a previous work [23], similar intra-cellular metal fractions (i.e., concentrations in EDTA washed leaves) were measured in the same three cardoon cultivars after 30 days of growth. The shorter exposure time chosen here to show genotoxicity [16], could have highlighted a peculiar trait (i.e., a faster metal accumulation capacity) of Sardo during the early growth stages. Moreover, this increase in element concentrations could be the consequence of a lower biomass production observed in a previous work in Sardo compared at least to Spagnolo [24].

The translocation factors (TFs) here calculated were all comparable to those calculated in previous experiments, suggesting that Cd and Pb translocation to shoot do not vary during plant growth. Moreover, a higher translocation for Cd compared to Pb, calculated here, was in agreement with previous literature reports [25,26,27]. According to our outcomes and considering that Pb translocation was one order of magnitude lower than Cd, we can confirm that genotoxic effect induced by Pb is more serious since it is exerted by a lower amount of Pb, compared to Cd. In agreement with our findings, several papers evidenced a genotoxic effect of Pb at very low concentrations both in vascular plants and even in cryptogams, e.g., [8,9,10,18,28]. In line with our results, Silva et al. [8] showed that Pb nitrate supplied in hydroponic cultures at similar concentrations as in the present experiment, induced genotoxicity in *Lactuca sativa*. By contrast, Kovalchuk et al. [29], investigating gene expression in plants of *Arabidopsis thaliana* L. chronically exposed to Cd and Pb, found a greater number of genes regulated by Cd, along with a higher genome instability of these plants, as well as higher metal-uptake compared to Pb.

Our findings confirmed that Cd and Pb entered the leaf cells of *Cynara cardunculus* var. *altilis*, cv. Sardo, Siciliano, and Spagnolo; however, it is also worth noting that, at parity of metal concentrations in the leaves of the three cultivars, a significantly lower fraction entered the cytoplasm (see concentration in EDTA washed shoots) in Spagnolo. Therefore, the lower genotoxicity observed in Spagnolo could be due to the lower intracellular metal concentration. In the present work, we highlighted that metal stress significantly decreased the size of epidermal cells in Spagnolo, thereby increasing their number and total wall surface. Based on the latter result, we hypothesize that these smaller cells could entrap a higher fraction of metals into their cell walls, which are well known as metal sequestration sites, e.g., [30]. Accordingly, the same cultivar, in the presence of Cd and Pb, developed more numerous and smaller stomata to preserve net photosynthesis efficiency under metal-stress [23].

Our results do not rule out other possibilities, for example, the set-up of more efficient mechanisms of DNA repair occurring in Spagnolo. Although it is reported that low concentrations of heavy metals could even stimulate some metabolic processes [31], metal-induced genotoxicity may have severe consequences to the life of a plant. In fact, high levels of metal-induced DNA damage may completely inhibit DNA repair mechanisms, leading to cell death [18,32]. However, specific experiments should be carried out to investigate DNA repair mechanisms in the three examined cultivars.

## 4. Materials and Method

### 4.1. Plant Material and Growth Conditions

Seeds of three cultivars of *Cynara cardunculus* var. *altilis* DC. (provided by Arca 2010 scarl), named Sardo (SAR), Siciliano (SIC), and Spagnolo (SPA), were germinated on wet filter paper for five days in the dark. Once primary roots and cotyledons were fully developed, 60 seedlings of each cultivar were moved to a hydroponic floating system, consisting of polystyrene plug trays floating on plastic tanks containing a constant volume of 5 L of aerated nutrient solution [33] at pH 5.5. Three tanks were used for the experimental plan: 20 seedlings of each cultivar per tank were exposed to a specific heavy metal or none as control, as follows; at two-true-leaf stage, either CdCl_2_ or Pb(NO_3_)_2_ at the concentration of 10 µM were supplied to the culture medium. The nutrient solution was renewed twice a week for 15 d. The parameters of the growth chamber were constantly monitored to maintain controlled conditions of temperature 24/18 °C, relative humidity (RH) 55–75% (day/night), and a photoperiod of 16 h light per day with a Photosynthetic Photon Flux Density (PPFD) at the top of the canopy of 180–200 μmol photons m^−2^ s^−1^. After 15 d culture, plants were collected and used for molecular and chemical analyses.

### 4.2. DNA Extraction and ISSR Amplification

Three plant shoots for each cardoon cultivar and treatment were collected and washed in sterile water. Total genomic DNA was extracted by using a modified CTAB (cetyl-trimethyl ammonium bromide) method [34]. DNA amplification was performed by using 20 different ISSR primers with anchor sequence (Table 3) selected after initial screening in a set of 40. Anchor sequence offers the advantage to avoid the sliding of the primer along the repeated motif, which would lead to the formation of products of different lengths in the same locus, giving rise to confusing results and an overestimation of the variability [15].

Polymerase chain reactions (PCR) were carried out three times for each sample to confirm the reproducibility of banding patterns and exclude possible artifacts. Only bands amplified in the three PCRs performed were selected for the analysis. The amplification reaction mixture consisted of DNA (15 ng), ddH_2_O, 10X Dream Taq Buffer, 1 mM MgCl_2_, 0.2 mM each dNTP, 0.6 mM primer, and 1 U Dream Taq DNA polymerase (Thermo Scientific).

The thermocycler (Esco Swift Maxi Thermal Cycler) was programmed for an initial denaturation step at 94 °C for 3 min, followed by 38 cycles with 1 min at 94 °C, 1 min annealing and 2 min extension, and finally one cycle of 7 min at 72 °C. Annealing temperatures, different for each primer, are reported in Table 3. Amplification products were separated by electrophoresis on 2% agarose gel with Tris-borate-EDTA (TBE) buffer stained with GelRed Nucleic Acid 10,000 (biotium) and visualized under UV light using GelDoc (Bio-Rad). Size estimates of the ISSR bands were made using a 100-bp ladder (Thermo Scientific) as a molecular marker.

### 4.3. Chemical Analysis of Plant Materials

To evaluate the total amounts of intra- and extra-cellular fractions of Cd and Pb, an EDTA washing was used. This step is part of the sequential elution technique [23,35,36,37] and moves away the metal fraction weakly bound to the plant tissues (i.e., the extracellular one), that includes the metal present in the intercellular space solution and the metal bound to the wall (i.e., the apoplast). What remains in the plant tissues after washing with EDTA represents, therefore, the intracellular fraction. Metal (Cd or Pb) treated samples (see Section 2.1) were divided into two batches: one batch, composed of 7 plantlets, was directly analyzed for the total element content in the roots and in the shoots separately, after dehydration and acid digestion. The shoots of the second batch (7 plantlets) were put in 20 mL 20 mM Na_2_EDTA solution for 20 min and then rinsed in deionized water, to remove the extracellular soluble fraction of Cd or Pb, i.e., the apoplastic fraction [35,36,37]. All samples were weighted and dried in the oven at 40 °C until constant weight, dehydrated and grounded in an agate mortar, and digested in 5 mL HNO3 65% (hyperpure, Carlo Erba) and 2.5 mL H_2_O_2_ 30% (Sigma Aldrich) for metal analysis.

The digested plant samples were diluted to 25 mL in Millipore water and subsequently filtered to analyze Pb and Cd by Flame AAS (Varian Spectra AA 220 FS). The reference plant material CTA-OTL-1 (oriental tobacco leaves) was also acid-digested and analyzed for Cd and Pb concentrations by Flame AAS, to determine the recovery percentages of the two elements; they were 96 and 107%, respectively. For calibration curves, Pb and Cd standard solutions were prepared in 0.1 M HNO_3_ by dilution of Pb(NO_3_)_2_ and Cd(NO_3_)_2_ stock solutions, respectively. Lead (II) and cadmium (II) nitrate stock solutions were prepared by dissolving lead or cadmium metal, 5N8 (Metal Research) with a nitric acid stock solution. The exact metal concentration in both solutions was determined by complexometric titration with EDTA.

### 4.4. Epidermal Cell Counting and Sizing

The abaxial surface at the mid lamina of two leaves per treatment and cultivars was covered by nail topcoat to obtain a leaf lamina replica to be observed under a light microscope. Images acquired were analyzed by ImageJ software (National Institutes of Health, MD, USA) to measure epidermal cell area for a total of 20 cells per cultivar and treatment (i.e., a total of 180 measurements). Moreover, epidermal cells were counted in other 20 images per thesis (Figure S1), for a total analyzed surface of about 93,000 µm^2^ × 20 = 1,860,000 µm^2^ = 1.86 mm^2^ for each treatment and cultivar.

### 4.5. Data Analysis

The datasets were normally distributed and homoscedastic; the normality and homogeneity of the variances of the datasets were assessed by the Shapiro–Wilk’s test and Levene’s test, respectively. Data elaborations and basic statistics were conducted by using Microsoft Excel. Statistical tests were performed by means of IBM SPSS Statistics for Windows (IBM Corp. Released 2020, Version 27.0. Armonk, NY: IBM Corp). ISSR profiles for each primer were analyzed by using the software GelAnalyzer 2019 for band counting and size/intensity assignment. For each lane, we considered the band profiles occurring in a range between 200 and 1300 bp; the software output was manually checked for further control. Changes in the ISSR patterns were expressed as a decrease in Genomic template stability (GTS), a qualitative measure of DNA stability based on ISSR banding of treated samples compared to the untreated samples. The GTS was calculated by the formula:

[1 − (n − a)/n] × 100(1)

where a is the average number of polymorphic bands in each treated sample and the number of all bands in the control. Firstly, genetic variation was estimated among three control plants, as they were plants from seeds, i.e., genetically different from each other. Then, one control plant was arbitrarily fixed as a reference control plant and its DNA profile was compared to the other two control DNA samples to quantify the GTS in control condition (i.e., in the absence of metal stress). The same control plant was used as a reference sample for comparison with DNA from metal-treated plant samples. Polymorphism in ISSR profiles included the disappearance and appearance of bands in comparison with the control. The differences in band intensity were also calculated by GelAnalyzer software in comparison with the bands of the ladder 100 bp (here used as gel marker), having known DNA concentrations; only difference of intensities higher than 20% were considered. The average GTS was calculated for each treatment and expressed as a percentage of the variable bands compared to the total band number. The polymorphism in ISSR profiles considering the presence/absence of bands and different band intensity is reported separately.

## 5. Conclusions

The present work investigated the genotoxic effect of heavy metals in three cardoon cultivars, Sardo, Siciliano, and Spagnolo in relation to element distribution between apoplast and symplast. In line with the literature reports, in all the cultivars, the genotoxic damage induced by Pb was more severe compared to Cd. However, a cultivar specific response was evidenced since Spagnolo showed, under metal stress, a significantly higher genome template stability compared to the other examined cultivars. This result can find a feasible explanation in the element distribution between apoplast and symplast; in the cultivar Spagnolo indeed, a limited amount of Cd and Pb represented the intracellular fraction (i.e., the fraction found in EDTA-washed leaves). In other words, the lower genotoxicity observed in Spagnolo could be justified by a lower heavy metal fraction in the symplast. Furtherly, morphological observation provided a possible mechanism to explain this cultivar-specific metal distribution. In fact, light microscopy observations highlighted that Spagnolo, under metal stress, developed smaller, and more numerous epidermal cells compared to control; these cells would provide a larger wall surface offering a wider metal sequestration compartment in the apoplast.

## Figures and Tables

**Table 1:** Element concentrations (mg kg^−1^, mean ± SD, *n* = 7) in shoot without washing (L), after EDTA washing to eliminate the apoplastic fraction (L+ EDTA; see par. 2.3), and root (R) in the three cultivars Sardo (SAR), Siciliano (SIC), and Spagnolo (SPA); the translocation factor (TF = (L)/(R)) was also calculated for the three cultivars. Different letters indicate significant differences (*p* < 0.05) according to Tukey’s test.

	SAR	SIC	SPA
**Cd**			
**L**	133 ± 9 c	111 ± 7 d	114 ± 8 d
**L + EDTA**	93 ± 9 e	79 ± 7 e	66 ± ef
**R**	482 ± 33 a	386 ± 32 b	420 ± 38 ab
**TF**	0.28	0.29	0.27
**Pb**			
**L**	46 ± 7 c	38 ± 5 c	43 ± 5 c
**L + EDTA**	31 ± 4 d	26 ± 5 de	22 ± 3 e
**R**	770 ± 65 a	652 ± 35 b	656 ± 36 b
**TF**	0.06	0.06	0.06

**Table 2:** Epidermal cell size (area, μm^2^) and cell number of *C. cardunculus*, cultivars Spagnolo (SPA), Sardo (SAR), and Siciliano (SIC) in control (Ct) and metal-treated samples (Cd and Pb); mean values ± standard deviations (*n* = 20).

		SPA	
	**Ct**	**Cd**	**Pb**
**Cell size**	2075 ± 264a	935 ± 223b	901 ± 236b
**Cell number**	81 ± 6.9b	147 ± 13a	145 ± 12a
		**SAR**	
	**Ct**	**Cd**	**Pb**
**Cell size**	2036 ± 211a	1994 ± 318a	1989 ± 220a
**Cell number**	79 ± 5.8b	78 ± 4.6b	78 ± 5.6b
		**SIC**	
	**Ct**	**Cd**	**Pb**
**Cell size**	2046 ± 331a	2061 ± 261a	1920 ± 313a
**Cell number**	77 ± 4.8b	78 ± 4b	77 ± 5.8b

Different letters indicate significant differences (*p* < 0.05) among the treatments according to Tukey’s post hoc test.

**Table 3:** Details of the twenty primers used and melting temperatures (Tm).

	Primer	Primer Sequence 5′-3′	Number of Bases	Tm (°C)
1	ISSR 10	AGAGAGAGAGAGAGYC	16	48
2	ISSR 14	TGTCACACACACACACAC	18	54
3	ISSR 15	GGTCACACACACACACAC	18	53.8
4	ISSR 18	GTGCACACACACACACAC	18	56
5	ISSR 19	CGGCACACACACACACAC	18	57.2
6	ISSR 20	CCTGCACACACACACACAC	19	56.9
7	ISSR 22	GTGCTCTCTCTCTCTCTC	18	50.8
8	ISSR 23	GAGTCTCTCTCTCTCTCTC	19	49.9
9	ISSR W814	CTCTCTCTCTCTCTCTTG	18	47.6
10	ISSR W898	CTCTCTCTCTCTRY	14	39.6
11	ISSR W899	CTCTCTCTCTCTRG	14	40
12	ISSR W901	GTGTGTGTGTGTYR	14	44
13	ISSR 8082	CTCTCTCTCTCTCTCTCTG	19	49.9
14	ISSR 8564	CACCACCACCACCACCACCAC	22	64.1
15	ISSR 8565	GTAACCACCACCACCACCACC	21	64.4
16	ISSR TE	GTGGTGGTGGTGRC	14	44
17	ISSR HAD	CTCCTCCTCCTCRC	14	44
18	ISSR MAN	CACCACCACCACRC	14	44
19	ISSR DAT	GAGAGAGAGAGAGARC	16	46
20	ISSR W843	CTCTCTCTCTCTCTCTRA	18	47.1

## Data Availability

All data are reported in the text and in Supplementary Materials.

## Supplementary Materials

The following supporting information can be downloaded at: https://www.mdpi.com/article/10.3390/plants11040475/s1, Figure S1: Epidermal cell count. Red spots indicated one epidermal cell; yellow spot indicate a stoma; Table S1: Changes of total bands in samples untreated (Ct) and treated with Cd and Pb compared to the control reference plant (C). Polymorphism in ISSR profiles based on band changes common to the three replicas: a—appeared band; b—disappeared band; c—increased band intensity; d—decreased band intensity. C-bands is the number of bands observed in the control reference plant with each primer. For more details see the Materials and Methods section.
