# Supplementary material for: Metals Induce Genotoxicity in Three Cardoon Cultivars: Relation to Metal Uptake and Distribution in Extra- and Intracellular Fractions
Source: Plants (Basel). 2022 Feb 9;11(4):475. doi: 10.3390/plants11040475 (PMC8876339; doi:10.3390/plants11040475)
Supplement: Supplementary file 1 [file plants-11-00475-s001.zip › SUPPLEMENTARY/figure S1.pdf]

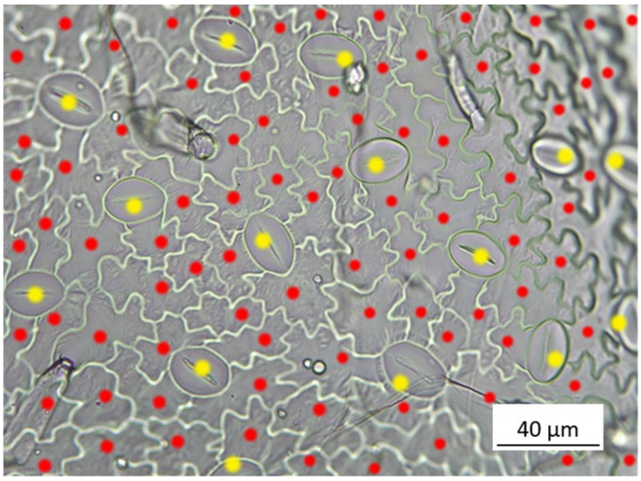

Figure S1. Epidermal cell count. Red spots indicated one epidermal cell; yellow spot indicate a stoma.
