# Supplementary material for: Metals Induce Genotoxicity in Three Cardoon Cultivars: Relation to Metal Uptake and Distribution in Extra- and Intracellular Fractions
Source: Plants (Basel). 2022 Feb 9;11(4):475. doi: 10.3390/plants11040475 (PMC8876339; doi:10.3390/plants11040475)
Supplement: Supplementary file 1 [file plants-11-00475-s001.zip › SUPPLEMENTARY/table s1.pdf]

Table S1. - Changes of total bands in samples untreated (Ct) and treated with Cd and Pb compared to the control reference plant (C). Polymorphism in ISSR profiles based on band changes common to the three replicas: a - appeared band; b - disappeared band; c - increased band intensity; d - decreased band intensity. C-bands is the number of bands observed in the control reference plant with each primer. SAR, SIC and SPA indicate the three cardoon cultivar analyzed: Sardo, Siciliano and Spagnolo. For more details see M&M.

**SAR**		**Ct**				**Cd**				**Pb**			
**Primer**	**C - bands**	**a**	**b**	**c**	**d**	**a**	**b**	**c**	**d**	**a**	**b**	**c**	**d**
**10**	8	2	2	2	0	5	1	4	0	5	0	6	0
**14**	7	5	1	0	0	8	2	0	0	17	1	4	0
**15**	12	1	0	0	0	11	14	3	0	7	11	0	5
**18**	12	1	2	2	0	7	15	3	0	11	11	2	0
**19**	14	4	2	0	2	4	5	0	5	6	7	0	4
**20**	11	0	0	0	1	8	4	0	3	9	2	0	5
**22**	9	1	2	0	0	2	4	3	0	4	2	8	0
**23**	10	3	3	0	0	8	6	1	2	9	5	2	0
**w843**	5	1	0	0	0	6	4	2	3	2	3	3	2
**w814**	5	0	1	1	1	1	3	2	3	3	3	6	1
**w898**	11	1	3	0	1	19	8	3	2	23	7	8	2
**w899**	8	0	0	0	0	0	9	0	0	7	6	0	2
**w901**	12	4	2	0	0	11	14	0	2	7	15	0	1
**8082**	7	2	2	0	0	0	3	5	0	1	5	6	0
**8564**	12	0	1	0	0	5	5	2	0	3	10	2	0
**8565**	14	3	3	1	1	2	6	3	0	0	8	3	3
**DAT**	9	0	1	0	0	2	8	1	0	4	8	5	0
**HAD**	7	1	0	0	0	4	3	2	3	8	2	3	0
**MAN**	10	1	1	0	0	1	3	0	0	3	6	1	0
**TE**	11	1	1	3	0	1	1	1	0	4	2	1	2
**Tot**	194	31	27	9	6	105	118	35	23	133	114	60	27
**a+b**		58				223				247			
**a+b+c+d**		73				281				334			

**SIC**	**Ct**	**Cd**	**Pb**

Primer	C - bands	a	b	c	d	a	b	c	d	a	b	c	d
**10**	12	0	2	2	0	3	5	0	1	7	5	2	3
**14**	11	0	0	0	0	9	8	4	0	5	5	4	1
**15**	8	0	0	0	0	5	0	0	0	1	2	0	0
**18**	11	2	3	0	0	4	3	1	3	5	6	1	2
**19**	14	0	0	0	0	0	0	0	0	0	0	0	0
**20**	18	0	0	0	0	0	0	0	0	0	0	0	0
**22**	8	0	4	0	6	0	5	8	8	2	3	0	12
**23**	10	0	0	0	0	5	4	0	4	5	5	1	2
**w843**	6	2	1	1	1	3	0	3	0	4	1	4	0
**w814**	5	2	0	3	1	4	4	5	1	8	4	6	1
**w898**	20	5	2	4	2	4	12	9	2	5	13	11	0
**w899**	4	0	0	0	0	0	3	2	0	2	2	1	0
**w901**	14	1	6	0	0	5	5	0	0	2	7	4	0
**8082**	6	3	4	0	0	5	5	2	0	2	4	1	0
**8564**	6	1	1	0	0	0	3	4	0	0	3	6	0
**8565**	9	1	1	2	0	3	4	3	0	4	4	2	0
**DAT**	7	2	0	0	3	0	1	3	0	0	1	2	1
**HAD**	13	5	8	2	0	6	16	5	0	3	13	11	1
**MAN**	9	3	3	0	0	6	4	0	0	3	2	4	1
**TE**	9	6	3	0	1	7	0	0	2	5	3	1	2
**Tot**	200	33	38	14	14	69	82	49	21	63	83	61	26
**a+b**		71				151				146			
**a+b+c+d**		99				221				233			

**SPA**		**Ct**				**Cd**				**Pb**			
**Primer**	**C - bands**	**a**	**b**	**c**	**d**	**a**	**b**	**c**	**d**	**a**	**b**	**c**	**d**
**10**	10	1	2	0	0	0	3	5	0	2	4	2	0
**14**	9	3	1	3	0	0	3	1	0	3	7	2	0
**15**	14	0	0	0	0	0	0	0	0	0	0	0	0
**18**	15	0	0	0	0	0	0	0	0	0	0	0	0
**19**	13	0	0	0	0	0	0	0	0	0	0	0	0

**20**	9	0	0	0	0	0	0	0	0	0	0	0	0
**22**	10	4	4	2	0	11	9	12	0	10	4	15	0
**23**	12	1	1	0	0	9	11	9	0	6	8	9	0
**w843**	10	5	6	0	0	10	4	3	1	6	8	5	1
**w814**	6	3	2	4	2	5	3	3	0	1	2	4	3
**w898**	20	0	0	0	3	0	2	4	2	1	3	3	3
**w899**	4	1	1	1	0	1	0	3	1	1	2	2	0
**w901**	9	0	0	0	0	0	0	0	0	0	0	0	0
**8082**	6	0	0	2	0	3	2	8	0	5	2	11	0
**8564**	4	2	2	0	0	3	2	0	0	3	3	0	0
**8565**	10	0	0	1	3	1	3	0	1	2	2	0	2
**DAT**	6	0	0	0	0	3	0	3	0	1	0	0	0
**HAD**	11	2	1	2	0	4	6	8	0	5	6	6	1
**MAN**	13	5	4	2	0	6	5	1	0	2	3	2	0
**TE**	15	0	0	0	0	0	0	0	0	0	0	0	0
**Tot**	206	27	24	17	8	56	53	60	5	48	54	61	10
**a+b**		51				109				102			
**a+b+c+d**		76				174				173			
